# Presence of an Isolated Hydatid Cyst in the Left Kidney: Report of a Case of This Rare Condition Managed Surgically

**DOI:** 10.1155/2016/6902082

**Published:** 2016-06-27

**Authors:** Daniel Paramythiotis, Petros Bangeas, Konstantinia Kofina, Vassileios Papadopoulos, Antonios Michalopoulos

**Affiliations:** 1st Propedeutic Surgical Department, AHEPA University Hospital, Aristotle's University of Thessaloniki, 54636 Thessaloniki, Greece

## Abstract

*Introduction.* Hydatid cyst disease caused by* Echinococcus granulosus* is rarely presented in the kidneys, whereas isolated renal occurrence is estimated to be about as low as 2–4% of all cases. We present a case of a female patient suffering from this condition that was treated successfully in our department.* Case Presentation.* A 44-year-old woman was incidentally diagnosed with a 14 cm left renal cystic mass through ultrasound imaging performed during upper abdominal pain investigation. Laboratory examinations were normal and CT imaging set the diagnosis of an isolated left renal hydatid cyst. The cyst was excised and the postoperative period was uneventful.* Discussion.* Isolated renal hydatid cyst is a very rare condition and could possibly be misdiagnosed with other renal masses. The clinical history, laboratory tests, and thorough radiologic imaging are crucial for the accurate preoperative diagnosis.

## 1. Introduction

Hydatid cyst disease is a parasitosis caused by* Echinococcus granulosus* [1] and is endemic especially in Eastern Europe, Middle East, Alaska, Canada, South America, Australia, and New Zealand [2]. The causative tapeworm lives in the small intestine of definitive hosts, such as dogs and other canids [3], whereas, when transmitted to humans, it can affect various organs, including the liver, lungs, brain, and the urinary tract [4]. The kidney is the most commonly affected organ of the urinary tract, although this involvement is rare [5]; however, isolated renal involvement is even rarer (2–4% of all cases) [6] and reports of this condition are limited. This benign disease can remain asymptomatic for many years, while hematuria or/and hydatiduria (presence of daughter vesicle in urine) occurs in 10–20% of all cases [7]. Diagnosis is usually made by clinical and radiological findings; however, diagnostic steps and complete and exact pathophysiology elucidation of urinary tract hydatid cysts are needed to be performed. Herein, we present a case of an asymptomatic female patient with a primary hydatid cyst in the left kidney that was treated successfully in our department.

## 2. Case Report

**Figure 1:**
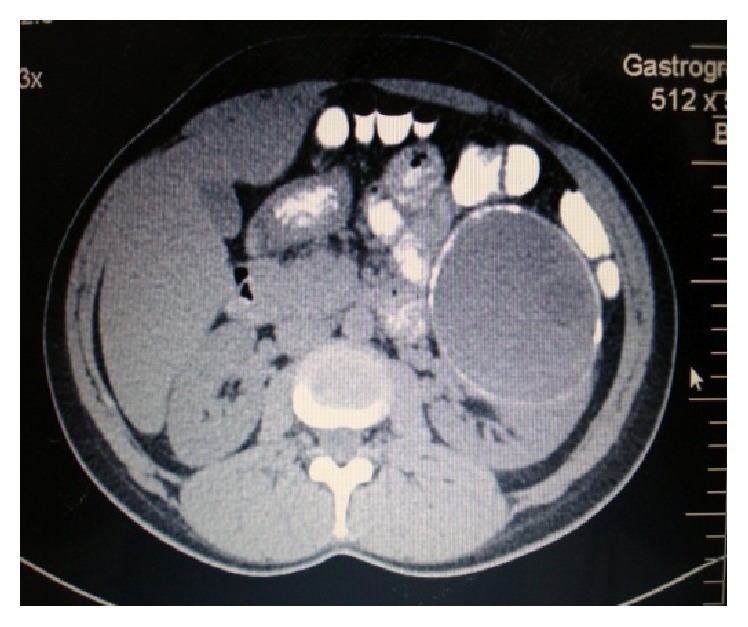
CT imaging revealing the 14 cm left renal cystic mass.

A 44-year-old woman presented in our surgical department complaining of the presence of chronic epigastric pain and mild fever. For these symptoms, radiologic imaging through upper abdominal ultrasonography had been performed and demonstrated a multicystic lesion in the left kidney. With this history, after admission, systemic clinical examination revealed a smooth, nontender mass in the left hypogastric region. Clinical examination concerning all the other systems revealed normal findings. Therefore, a contrast-enhanced computed tomography (CT) of the upper abdomen was performed, which confirmed the ultrasonography data (Figure 1), displaying a hypodense multicystic mass with calcified thick walls, which is a characteristic of hydatid disease.

The laboratory findings, including liver and renal function tests, were within normal limits. Immunological examination revealed elevated* Echinococcus* antibody titers (IgG levels: 2.50 – positive values >1.1). Urine tests did not reveal any signs of hydatiduria. Tumor markers as CEA, CA19-9, and AFP were all within normal limits, as well.

**Figure 2:**
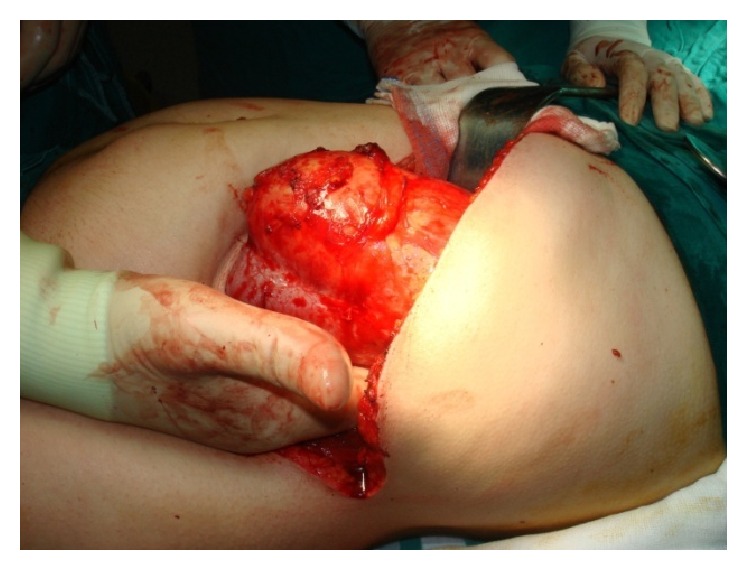
Access to the left kidney through flank approach.

**Figure 3:**
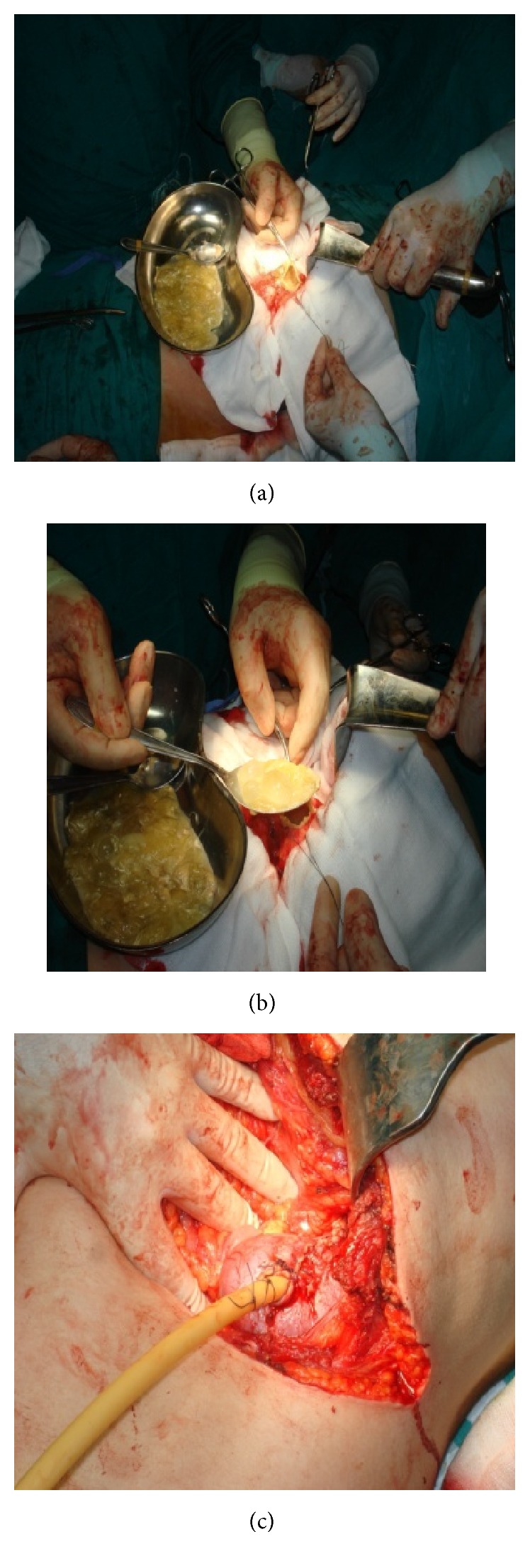
Intraoperative removal of the cyst's content and drain placement.

**Figure 4:**
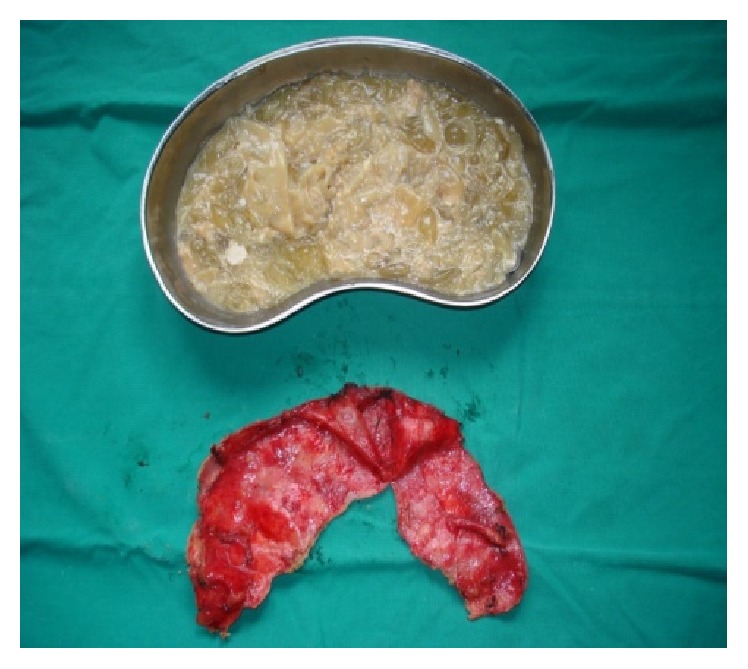
Surgical specimen, content, and walls of the hydatid cyst.

Transabdominal approach through subcostal incision was performed (Figure 2). Abdominal exploration revealed the renal hydatid cyst and open cystectomy was performed (Figures 3(a), 3(b), 3(c), and 4). No other intra-abdominal pathology was noticed, as well. The postoperative period was uneventful. After a 6-month follow-up period, the patient was asymptomatic and doing well.

## 3. Discussion

Hydatid cyst disease is a zoonosis caused by the larval stage of the parasite* Echinococcus granulosus*, a member of the order Cestoda, the family Taeniidae. The eggs of the parasite are excreted in the feces of the host (usually dogs and foxes). Humans get infected through contact with the definitive host or ingesting contaminated soil, water, and vegetables [3]. The eggs transform to larvae in the human's digestive system; the larvae migrate into the small bowel wall and the mesenteric circulation, getting filtered by the liver, which is the reason why hepatic infection is so common. The second site of filtration is the lung, which is involved in 15% of the cases [7]. However, almost every body tissue can be infected through hematogenous dissemination [8]. Kidney involvement represents 4% of all cases and is rare compared to that in the liver or lung, even more as an isolated site of infection [5].

Symptoms and signs of hydatid cyst disease depend on the involved organ, the site, and the secondary spread, while a palpable mass is the most common clinical finding. The patients with renal hydatid cyst usually present with vague pain in the lumbar or flank region [9]. The rupture of the cyst into the urinary collecting system causes hydatiduria and is a pathognomonic sign of renal hydatidosis, a finding that is present in only 10–20% of the cases. Gross hydatiduria is uncommon but diagnostic of the condition.

Differential diagnosis of hydatid cysts of the kidney from other space-occupying renal masses can be challenging. However, the combination of factors such as the slow increase of growth, the characteristic imaging findings, and serological test results can be revealing [9].

Eosinophilia is noted in about 50% of cases [8]. Serological tests in primary renal hydatidosis are usually negative. Radiologic imaging through ultrasonography (US), computed tomography (CT), and magnetic resonance imaging (MRI) proves the diagnosis. Ultrasound (US) can be helpful in the diagnosis of benign tumors (simple cysts). It can also be used as a practical guide during interventional procedures. In CT imaging, the mass is demonstrated to be uniloculated or multiloculated with low attenuation, surrounded by a thick wall, whereas in MRI the wall is hypointense in T2-weighted sequences and the content shows a characteristic water signal intensity in both T1 and T2 [10, 11].

Surgery is the usual treatment of option through an open approach [12], whereas retroperitoneal approach is preferred to avoid further contamination of the peritoneal cavity [13]. Also to avoid the spread of the disease, the cyst should be removed without rupture. When possible, kidney sparing cyst removal is performed through cystectomy and pericystectomy; however, nephrectomy is needed when the hydatid cyst invades a major renal part or in cases of hydatiduria [14]. Perioperative chemotherapy with albendazole is considered useful for the prevention of further localizations [15]. Intraoperative use of hypertonic saline injected in the cyst can exterminate the infective daughter cysts [8].

In our case, we considered feasible a transabdominal approach through subcostal incision. Abdominal exploration revealed the hydatid renal cyst and open cystectomy was performed. The kidney was reserved, because the hydatid cyst did not invade a major renal part and renal function tests were normal. Intraoperative use of hypertonic saline was decided in order to exterminate any infective daughter cysts. The postoperative period was uncomplicated and the patient received albendazole for three months. Therefore, after a 6-month follow-up period, the patient was asymptomatic and in a good health condition.

## 4. Conclusion

A limited number of isolated renal hydatid cysts are reported in the literature, while the disease is often misdiagnosed as a simple lumbar pain or a malignant renal mass. Careful diagnosis and complete pathophysiology of urinary tract hydatidosis are needed to be clarified. The clinical history, laboratory tests, and thorough radiologic imaging are crucial for the accurate preoperative diagnosis.
